# Treatment of Visceral Transplant Pseudoaneurysms Using Physician-Modified Fenestrated Stent Grafts: Initial Experience

**DOI:** 10.1007/s00270-019-02168-y

**Published:** 2019-02-06

**Authors:** Sebastian Mafeld, Jennifer A. Logue, Steven Masson, Rohan Thakkar, Aimen Amer, Colin Wilson, Gorab Sen, Derek Manas, Steven White, Robin Williams

**Affiliations:** 1 Interventional Radiology, Freeman Hospital, Freeman Road, Newcastle upon Tyne, NE7 7DN UK; 2 Hepatobiliary Surgery, Freeman Hospital, Freeman Road, Newcastle upon Tyne, NE7 7DN UK; 3 Hepatology and Liver Transplantation, Freeman Hospital, Freeman Road, Newcastle upon Tyne, NE7 7DN UK

**Keywords:** Anastomotic pseudoaneurysm, Physician-modified fenestrated stent grafts, Visceral transplant pseudoaneurysms, Endovascular pseudoaneurysm repair

## Abstract

Pseudoaneurysms after visceral transplantation represent a significant risk to patients. We report the successful treatment of three transplant (pancreas, liver and kidney) artery anastomotic pseudoaneurysms using physician-modified fenestrated endovascular stent grafts. In all cases, surgical repair was considered high risk and would have compromised the arterial supply to the graft. The endovascular approach in all cases obviated the need for surgical intervention and maintained graft arterial supply.

## Introduction

Pseudoaneurysms of the arterial anastomosis to visceral transplants are rare. They are more common in pancreas transplants because of sepsis and damage from pancreatic leaks with a reported incidence of up to 8% [1]. Their treatment remains controversial, but includes conservative, surgical and endovascular options. Open surgical repair is normally advocated, but more recently, with the evolution of endovascular practice, a greater variety of management options are available. Preservation of graft arterial supply is critical to preserve transplant function. We present three cases in which physician-modified fenestrated stent grafts were created and deployed successfully thereby excluding the pseudoaneurysm from pressurisation and maintaining graft vessel patency.

## Technique Overview and Case Descriptions

A stent graft for the treatment of a visceral transplant pseudoaneurysm is an off-label use that requires specific preoperative consent. Computed tomography (CT) angiographic imaging is essential for planning, and device modification should take place in a sterile environment. We recommend that it is done immediately prior to implant, in the same theatre, to minimise the risk of contamination through handling. The choice of stent graft device is pragmatic, determined by local availability and the size of artery to which the transplant is anastomosed. Thoracic aortic, abdominal aortic and iliac devices can be used.

The graft design is planned from the CT imaging in a similar manner to planning a standard complex EVAR. After a suitable donor stent graft has been identified, the rotational orientation and the distance from the proximal edge of the stent graft for the centre point of the fenestration should be measured from multi-planar reconstruction of the CT scan. 1-mm slices are generally the maximum slice thickness that we would consider sensible. The fenestration size is determined by the size of the transplant vessel and should be roughly equal.

**Fig. 1:**
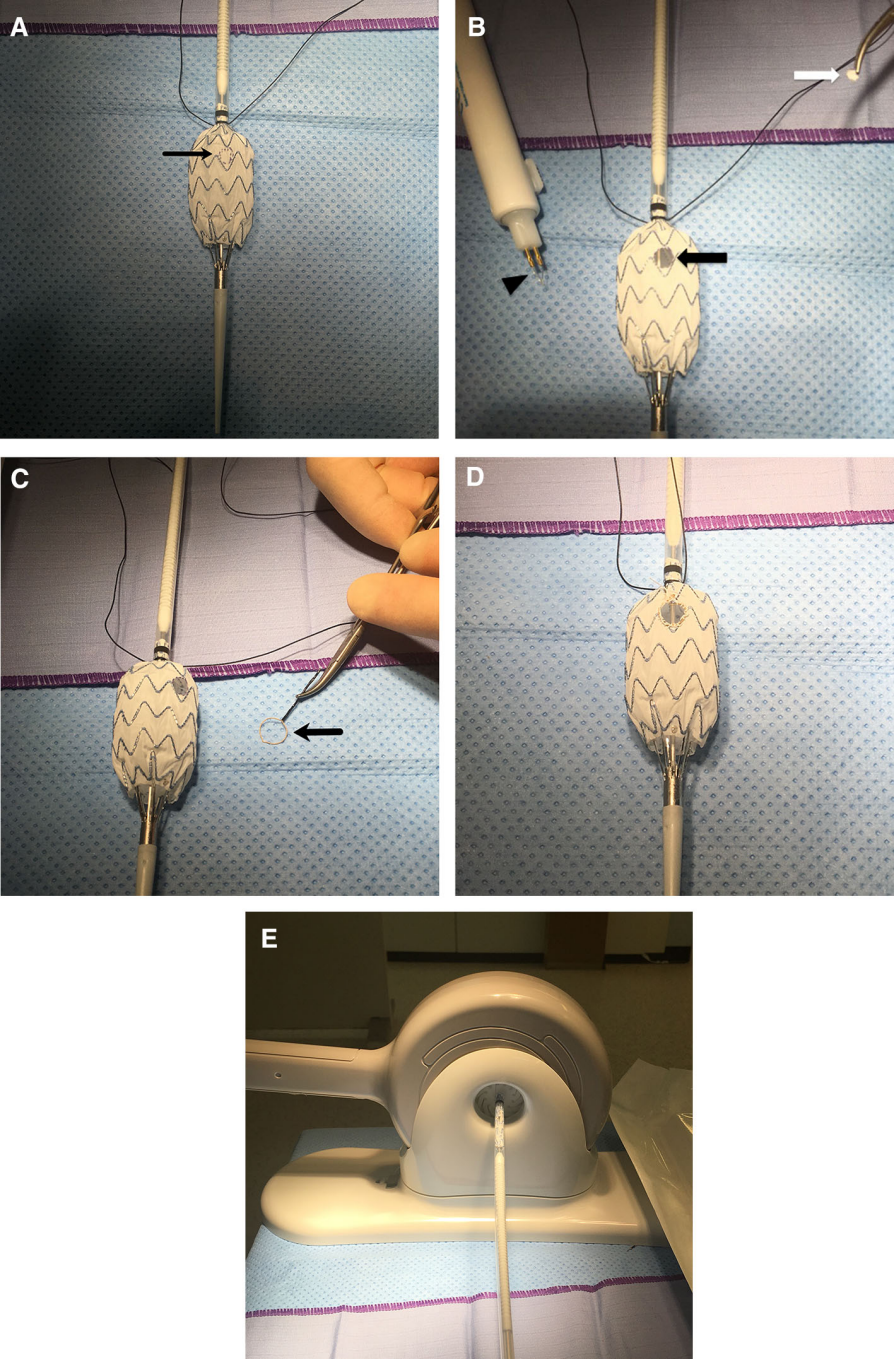
**A** Partial deployment of a stent graft in a sterile environment with marks drawn for the planned fenestration (arrow). **B** Accutemp low-temperature cautery pen (arrow head) used to create the fenestration (black arrow), with a segment of the stent graft removed (white arrow). **C** Gooseneck snare (arrow) used to reinforce the fenestration. **D** The snare has been sutured in place around the custom fenestration using PTFE sutures. **E** A valve stent crimper is used to re-package the stent graft into the delivery system

The graft is typically deployed on a sterile field (Fig. 1A), and the planned site for the fenestration is drawn with a sterile pen. The simplest way to determine the rotational position of the fenestration is to convert the angle to a linear measurement on the circumference of the graft. With the stent graft temporarily flattened, each side of the graft represents ½ of the circumference.

**Fig. 2:**
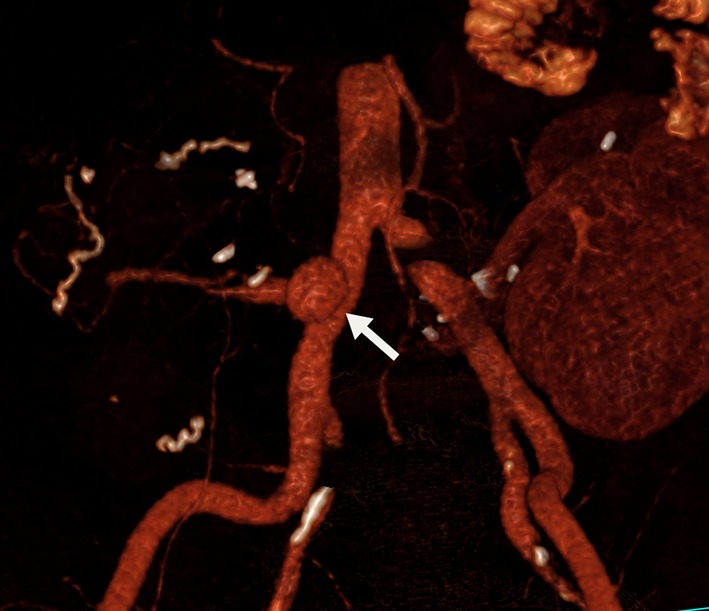
3D reconstructed CT demonstrating a 14-mm pseudoaneurysm (arrow) arising from the right common iliac artery at the origin of the Y graft arterial conduit

**Fig. 3:**
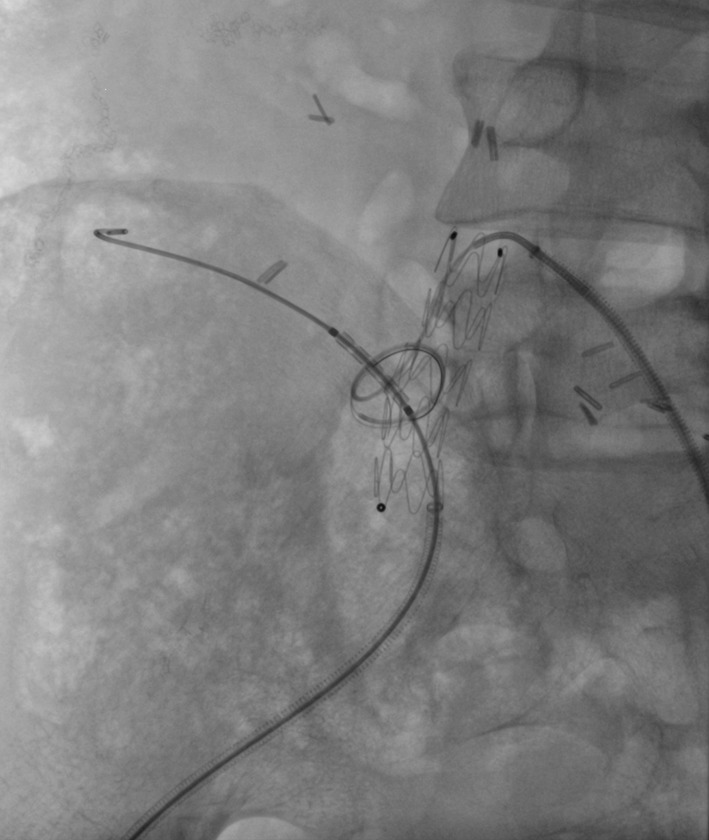
Physician-modified fenestrated stent graft deployed in the right common iliac artery. Fenestration catheterised with guidewire in the pancreatic transplant artery. Atrium V12 balloon mounted stent in place and ready to be deployed

Metal struts from the skeleton of the stent graft should be avoided, and in most cases it is possible to position a single fenestration away from the struts; however, in other more complex designs, we have successfully removed struts with wire cutters, carefully suturing over the cut ends.

Existing radiopaque markers are useful to orientate the graft, and one marker on the proximal edge should be used to mark either zero degrees or the fenestration angle, depending on user preference. If no markers are correctly positioned, they can be removed and re-sutured onto a more useful position.

Polyester donor stent grafts can be easily shortened with an Accutemp low-temperature cautery pen (Beaver Visitec International, Waltham, MA, USA). This pen is also used to create the fenestration (Fig. 1B). The fenestration is then reinforced with a gold gooseneck snare loop cut from the wire shaft (Fig. 1C). We have used either 7 mm or 10 mm microsnares (EV3 (Medtronic), Dublin Ireland). The 10-mm snare has better fluoroscopic visibility and can be reduced in size as needed. At least 5 mm of the snare shaft should be left attached to prevent the snare from unravelling. The snare is sutured in place around the custom fenestration using 5/0 PTFE sutures (Gore & Associates, Newark, Delaware, USA) (Fig. 1D). The modified device is then re-packaged into the delivery system. This is aided by the use of a valve stent crimper (Edwards Lifesciences, California, USA) (Fig. 1E) but is not essential. Alternative methods include wrapping a spiral of heavy suture or tape around the graft and unwrapping the suture as the graft is recovered by the delivery system outer sheath by an assistant. Great care should be taken to avoid graft twist. Finally the graft can be soaked in Rifampin to reduce the chance of infection [2].

Deployment is similar to a standard fenestrated EVAR. Accurate positioning of the fenestration during initial deployment is the key, particularly as there is only limited ability to move the main stent graft when it is uncovered. We recommend visualising the ostium of the target (transplant) artery in the two orthogonal planes which can be determined from the CT scan. The fenestration should be positioned adjacent to the target vessel, but the gooseneck snare is always deformed within the delivery system and does not give a reliable indication of rotational alignment, so the radio-opaque markers on the proximal edge of the stent graft should be used to finesse the rotation as the graft is initially uncovered. It can be helpful to position a catheter or guidewire within the target vessel to aid alignment.

Once the main graft is deployed, the fenestration should be catheterised from within the graft in a standard manner. A guidewire is passed into target vessel, followed by a long vascular sheath. An appropriate balloon mounted covered stent (e.g. Atrium Advanta V12) (Maquet, Rastatt Germany), sized to the transplant artery, is then deployed across the fenestration into the target vessel. The covered stent is over-dilated, typically to 8 to 12 mm within the fenestration to seal the fenestration and prevent migration.

The technique described herein was used in three transplant cases: pancreas, liver and kidney (summarised in Table 1). In all cases, surgical repair was considered high risk and could have compromised the arterial supply to the graft; therefore, endovascular management was preferred.

**Table 1:** Case summaries

Case	Gender	Age	Transplant	Pseudoaneurysm details	Procedure	Follow-up
1	Male	46	Pancreas as part of simultaneous pancreas and kidney (SPK transplant)	14 mm wide necked (8 mm) pseudoaneurysm arising from the right common iliac artery at the origin of the Y graft arterial conduit (Fig. 2)	Medtronic Endurant 13 mm × 80 mm stent graft limb extension (ETEW 13 13 c 80 EE) was shortened to 50 mm Single fenestration measuring 6 mm was created and reinforced with a 7-mm microsnare tip cut from the wire shaft, and sutured in place with 5/0 PTFE. Radio-opaque markers salvaged from the discarded portion of the stent graft were reattached with 7/0 prolene Fenestration catheterised (Fig. 3) and stented with 8 mm × 18 mm lifestream (Bard, Georgia, USA) balloon expandable covered stent flared to 10 mm	11 month follow up CT showed a preserved graft pancreatic artery supply with complete involution of the pseudoaneurysm (Fig. 4)
2	Male	37	Liver	5-cm saccular pseudoaneurysm at the origin of the hepatic arterial conduit	7-mm fenestration was created in a Medtronic Endurant stent graft cuff (ETCF 28 28 c 50 EE) Reinforced with a 10-mm gooseneck snare (reduced to 7 mm diameter) and sutured with 5/0 PTFE Atrium Advanta V12 covered stent (5 × 38 mm) deployed through the fenestration and flared to 10 mm	Migration of covered stent resulting in pseudoaneurysm expansion at 1 month with a presumed type III endoleak. A further 5 × 38 mm V12 stent was deployed and flared to 12 mm. CT at 2 weeks confirmed pseudoaneurysm exclusion and ultrasound at 6 weeks demonstrated preserved arterial supply to the liver
3	Male	45	Kidney	Anastomotic pseudoaneurysm compressing the transplant renal artery (Fig. 5), markedly reducing flow	Single fenestration was created in a Medtronic Endurant stent graft limb and reinforced with a 7-mm microsnare then sutured with 4/0 PTFE. Fenestration catheterised and stented with an Atrium Advanta V12 covered stent	Received 6-month dual antiplatelet therapy (Clopidogrel and Aspirin). Pseudoaneurysm successfully excluded with no recurrence at 12-month follow-up

**Fig. 4:**
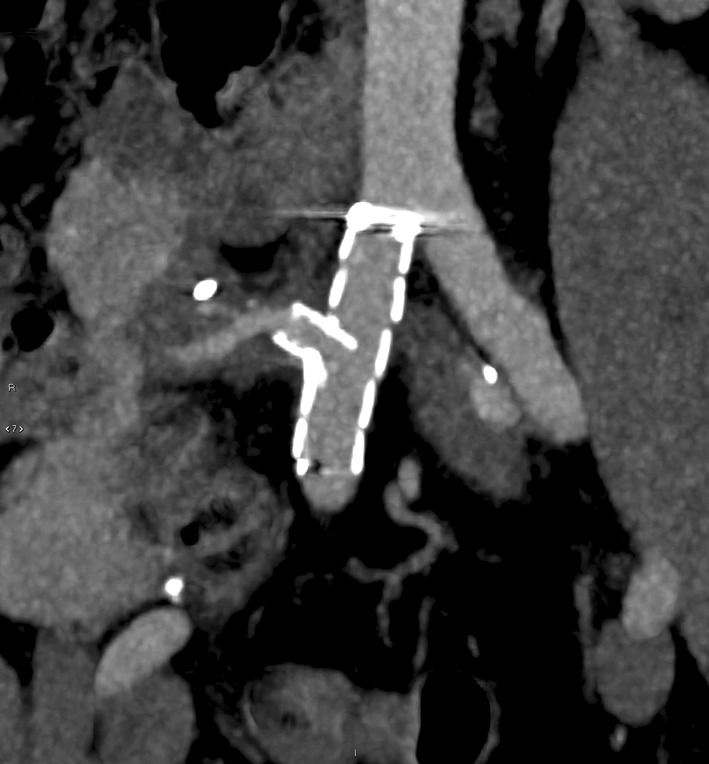
Contrast enhanced CT at 11 months demonstrating a patent graft pancreatic artery and involution of the pseudoaneurysm

**Fig. 5:**
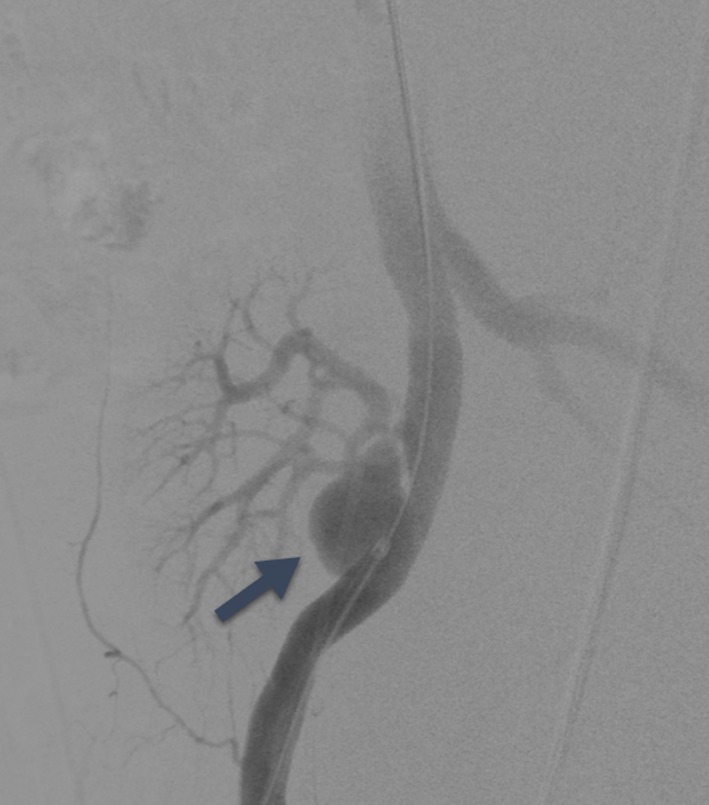
Iliac angiogram demonstrating a pseudoaneurysm (black arrow) arising from the transplant renal artery anastomosis. Also present (not shown) was a tight proximal renal artery stenosis

## Discussion

Visceral transplant anastomotic pseudoaneurysm formation is rare but a life- and transplant organ-threatening complication. They have a multi-factorial aetiology including surgical technique, infection, post-operative anastomotic/bile leak and pancreatitis [3, 4]. Clinical presentation is also highly variable ranging from asymptomatic (detected on routine imagine follow up) to life threatening (acute rupture) [5, 6]. Transplant hepatic artery pseudoaneurysm formation is estimated to occur in 0.27–3% of cases [5, 7–12]. The treatment of transplant pseudoaneurysms remains controversial, and due to its infrequent occurrence, there is a paucity of published literature to support a definitive management approach, where possible, open surgical revision of the anastomosis would normally be considered the first-line treatment. However, this often results in loss of the transplant. Transplant artery ligation is another management approach that has been suggested but again usually has the same outcome with gradual graft loss [6].

Endovascular management can be considered an alternative to open surgery in the management of transplant arterial pseudoaneurysms; however, its technical validity has been questioned [12]. Global experience remains limited with evidence restricted to case reports or small series. The location of transplant pseudoaneurysms is critical when considering an endovascular approach. For pseudoaneurysms of the transplant artery itself away from the anastomosis, stent grafting has been shown to be a successful technique [13–18]. However, when a transplant artery pseudoaneurysm involves the anastomosis or ostium, standard stent grafting is of limited value because it would involve sacrificing/covering the transplant vessel origin thereby risking the graft. In order to preserve flow into a transplant artery whilst excluding the pseudoaneurysm from blood flow, this requires stent grafting in a T configuration using a fenestration to preserve the graft vessel. There is no off-the-shelf, commercially available endovascular device designed for this purpose. Endovascular options are therefore limited to commercially made custom grafts or physician modification of existing off-the-shelf equipment. As commercially made custom endovascular stent grafts typically take 2–4 months to manufacture, this is not normally a suitable option to manage the emergent nature of transplant pseudoaneurysms.

The use of physician-modified fenestrated stent grafts has been described in the emergent treatment of complex aortic aneurysms with favourable clinical outcomes [19–22]. This served as a rationale for using this technique in the treatment of the transplant pseudoaneurysms presented in this series. An alternative endovascular approach that has been described in the context of an anastomotic pseudoaneurysm in a renal transplant is parallel grafting [23]. This involves two stent grafts deployed side by side, one into the transplant artery and one extending across the vessel origin. While this can exclude a pseudoaneurysm, it is a comparatively less attractive option as blood can continue to flow in the gutters between the stents thereby allowing continued perfusion to the pseudoaneurysm. The relative diameters and lengths of the involved vessels are important in determining suitability for parallel grafting. None of the presented cases were suitable for parallel grafting.

The aetiology of pseudoaneurysm formation in the cases described remains uncertain, but endovascular management has obviated the need for surgery thus far and maintained graft vascular supply. Some of these pseudoaneurysms developed in the presence of infection. This can be considered a contra-indication to endovascular repair, but there are reports of successful endovascular stent grafting in the presence of infection, either as a bridge to definitive open repair or as a definitive solution itself [24–26]. In the first case, infection was considered possible, but no clinical or micro-biological evidence was found. The patient remains well without antibiotics. The second case is perhaps more contentious. There is a significant possibility that the anastomotic failure was due to local fungal infection. The patient remains on anti-fungal therapy, and it is recognised that there is a significant possibility that further surgery will be required. However, neither the local transplant nor vascular surgeons felt that open repair of the pseudoaneurysm would be possible without loss of the arterial supply to the transplant liver and the recipient may still require a third liver transplant. In the third case, infection may have been the cause given the positive urine cultures for Klebsiella. The patient did receive treatment with Aztreonam/Dapsone and nevertheless remains well with a functioning renal transplant.

## Conclusion

Transplant pseudoaneurysms are a difficult to manage and high-risk patient group. The cases presented here using physician-modified fenestrated stent grafts successfully treated the pseudoaneurysms and maintained graft vascular supply. This technique is technically demanding but promising. Longer-term follow-up is needed to better understand this technique’s true clinical value.
